# Supplementary figures and images for: The Goto Kakizaki rat: Impact of age upon changes in cardiac and renal structure, function
Source: PLoS One. 2021 Jun 24;16(6):e0252711. doi: 10.1371/journal.pone.0252711 (PMC8224913; doi:10.1371/journal.pone.0252711)

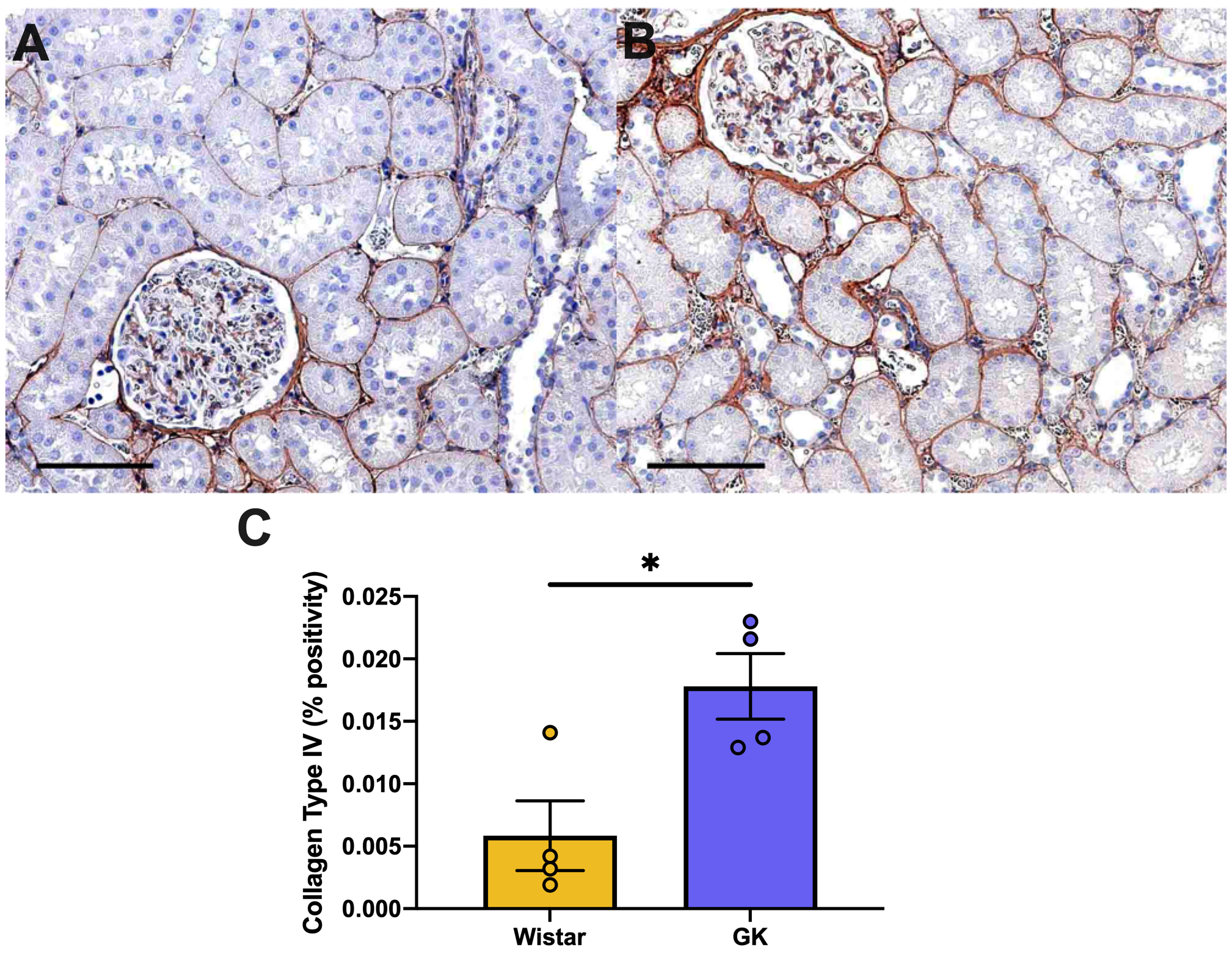

Supplement: S1 Fig — Representative Collagen IV stained Wistar (A) and GK (B) kidneys at 48 weeks, respectively. Percent positivity staining (C) * = p<0.05 when compared to Wistar rats; (students t-test was used to assess statistical significance). Yellow = Wistar and Blue = GK. Data is expressed as mean±SEM N = 4–8 per group. Scale bars 100 μm. (TIFF) [file pone.0252711.s001.tiff]
