# Supplementary material for: The Goto Kakizaki rat: Impact of age upon changes in cardiac and renal structure, function
Source: PLoS One. 2021 Jun 24;16(6):e0252711. doi: 10.1371/journal.pone.0252711 (PMC8224913; doi:10.1371/journal.pone.0252711)
Supplement: S1 Table — (DOCX) [file pone.0252711.s002.docx]

**S1 Table. Top five functional networks associated with overexpressed proteins identified in GK nuclear and cytosol proteomes.**

**
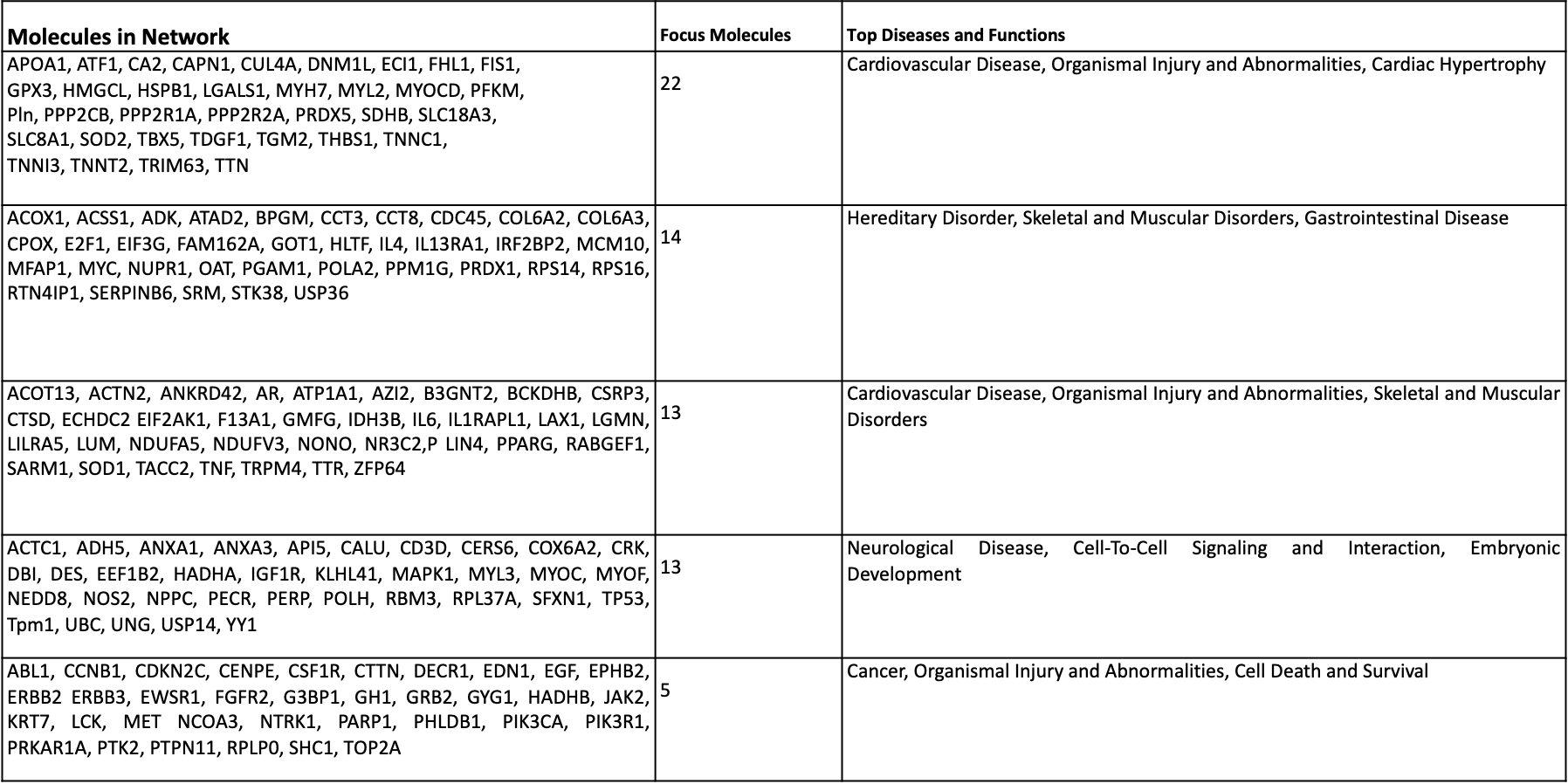
**
