# Supplementary material for: The Goto Kakizaki rat: Impact of age upon changes in cardiac and renal structure, function
Source: PLoS One. 2021 Jun 24;16(6):e0252711. doi: 10.1371/journal.pone.0252711 (PMC8224913; doi:10.1371/journal.pone.0252711)
Supplement: Attachment — Submitted filename: PLOS one comments.docx [file pone.0252711.s005.docx]

Dear Dr., Lionetti,

Thank you for giving us the opportunity to submit a revised draft of the manuscript “The Goto Kakizaki rat: Impact of age upon changes in cardiac and renal structure, function.” for publication in PLOS ONE. We greatly appreciate the time and effort that you and the reviewers committed to providing us with feedback. The critiques provided helped us immensely to improve the quality of our manuscript. We have incorporated the suggestions made by yourself and the reviewers. All changes and alterations are highlighted in the manuscript with track changes. Please see below a point-by-point response to the reviewers’ comments and recommended revisions, with the line numbers as to their location where applicable. Further we have uploaded additional supplementary material for the proteomics study.

Sincerely,

Patrick Meagher and Kim Connelly

Additional Editor Comments:

The authors should discuss their data in light of recent study demonstrating that ADAMTS 13 deficiency may underlie the onset of lethal arrhythmias in diabetes affecting lifespan (Diabetes. 2018 Oct;67(10):2069-2083.).

We thank the editor for this important comment. We politely ask that whilst this is important, it is outside the scope of the current paper as we did not explore the development of development of arrythmias but the onset of cardio-renal syndrome. We therefore humbly request that this not be included in the revised version of our paper.

Reviewer 1

Major:

1. One of the main conclusions of the manuscript is that the GK rat is not a good model to study cardiorenal phenotype. However, as described by the authors and others, this strain has a number of futures of both cardiac and renal disease progression. Therefore, it is suggested to focus on the discussion of the data and lessen this conclusion about the inapplicability of this model for this type of study.

Thank-you for this important insight. This was not our intention and we have altered our main conclusion – see page 26, line 957-963.

By 48 weeks of age, the diabetic GK rat demonstrates evidence of preserved systolic function and impaired relaxation, along with cardiac hypertrophy, in the presence of hyperfiltration and elevated protein excretion. These findings suggest the GK rat demonstrates some, but not all features of diabetes induced “cardiorenal syndrome”. This has implications for the use of this model to assess preclinical strategies to treat cardiorenal disease and may provide a model that best replicates the early complications of cardiorenal syndrome.

2. Renal function, as described in Figure 4 and the text, was tested at 40 weeks age. However, as described for protocol 1, animals were sacrificed at ~48 weeks. Which one is correct?

Thank-you we have corrected this to 48 weeks . Page 10 Line 229

3. It appears that kidney sections are available, and kidney weights to body weights were significantly different between groups. It would be nice if the authors check fibrosis.

This is an excellent suggestion; we have analysed Collagen IV content in paraffin embedded kidney sections. We observed an increase in Collagen IV content in GK rats compared to Wistar rats. This has been incorporated in the text, page 16, line 85 and supplemental figure 1:

Further, immunohistochemical staining of Collagen IV demonstrated Collagen IV content was significantly increased in GK rats compared to Wistar rats (P<0.05) at 48 weeks (supplemental figure 1).

4. Proteomic analysis: there is some inconsistency in the description of figure 9 and the numbers shown in the figure. As mentioned in the text, 163 proteins were uniquely overexpressed in HG cultures (Figure 9A), and 253 proteins are significantly overexpressed in GK rats (figure 9B), but boxes in the figure indicate 128 and 253 proteins, respectively. Furthermore, it should be important to compare the data in cell culture and in GK/Wistar rats to show overlap, if any. Please also describe key proteins/pathways identified by proteomic analysis rather than reference to supplemental table, which is also incomplete.

Apologies we have rectified this in the abstract please see page 20 line 823

However, 128 proteins where uniquely overexpressed in HG cultures compared to that of the NG Cultures (p<0.05, Figure 9A).

Please also see page 20 line 831-836

The top-enriched networks based on a high percentage of focus proteins in our datasets included pathways linked to cardiovascular disease. cardiac hypertrophy, cancer, cell death and survival with upregulation of proteins such as MYH7, MYL2, APOA1 and Phospholamban (Figure 9, Table of Top enriched networks from core analysis of both H9c2 and GK overexpressed proteins Supplemental Table 1).

Finally, we did compare the HG H9C2 cells and GK rats to see if there was overlap in pathways that were commonly upregulated between them. Please the additional supplementary files which have our proteomics analysis within them.

Please see page 20 line 836-838

Commonly upregulated proteins in both H9c2 cell lysates and GK hearts were involved pathways that regulated oxidative phosphorylation, Kreb’s cycle, mitochondrial dysfunction, beta-oxidation, and PI3K/Akt signalling (all p<0.05).

5. It would be great if the authors perform proteomic analysis of renal tissue as well. While it might be outside of the scope of this manuscript, this data will be important considering that the authors attempt to study cardiorenal interaction in GK rats.

This is a pertinent comment. The exploration of the kidney proteomics is outside the scope of the current study. Further, simple exploration of proteomics in kidney samples would likely have confounding results due to the differential protein expression between the medulla and cortex, owing to the complex nature of renal anatomy. Therefore, exploration of protein expression within these sections of the kidney would be extremely difficult to examine in whole kidney samples. We will pursue this in future studies, however.

6. The authors describe in the text (including abstract) that rats are age and sex matched. While this is correct, it is slightly misleading since only male rats are studied. This is especially critical since there is a significant difference between the development of diabetes between male and female.

Apologies, we have amended this to make it clear we are studying male sex.

See Abstract page 2 Line 33 and page 7 line 148

7. Progression of kidney disease of GK (compared to Wistar and T2DN rats, which were developed from GK rats) was recently described (PMID: 31566426). Earlier studies also described renal disease in GK and T2DN rats (PMID: 24319624). Consider comparing the findings and discuss if T2DN rats might be a better model to study cardiorenal phenotype.

Thank-you for the suggestion. We have made a comment on the T2DN rat, and provided appropriate reference to these two papers. see page 23 Line 591

Our current observation is in line with other observation within the literature which, suggest the GK rat has relatively mild phenotype of diabetic kidney disease (DKD) (PMID: 24319624, PMID: 31566426). The development of the T2DN rat, a sub-strain of the GK rat has shown much promise as it exhibits many of the hallmarks of clinical human DKD. However, the GK rat may be of use to understand the early changes which result in DKD and potentially contribute to cardio-renal syndrome.

8. Tables 1 and 2 – Include a number of rats for each group in the table.

Apologies. This has been corrected. See Table 1 - Page 12, Table 2 Page 16.

9. Figure 2 legend (page 14). It appears that letters referencing to wrong images “Where hearts of Wistar (A,C,E,G) rats and Goto Kakizaki (B,D,F,H) rats at early(28 weeks; A,B,E,F) and late (48 weeks;C,D,G,H)….“. The same comment about figure 6 (figures 6F-J).

Apologies. This has been corrected. Page 15 Line 348-353 & Page 18 Line 427-429

Minor:

1. Figure 2: A-D – Scales (lines/font) should be bigger. E and J: Describe a color for Wistar/GK rats.

Apologies. This has been corrected. Scale bars have been altered see new uploaded figure 2. The addition of a colour description has been added to figure legend.

2. Figure 2J. Is it statistical difference between Wistar and GK rats at 28 weeks?

Unfortunately, there was no significance between these groups.

3. Figure 4A and B – add scales.

Apologies. This has been corrected. Please see new figure 4.

4. Methods: Please describe what is a standard chow.

Apologies. This has been corrected. see methods, page 7 line 153.

For more info on the rodent diet, the vivarium orders 18% protein diet which can be found here: https://www.envigo.com/rodent-natural-ingredient-2018-diets

5. Please mention that 40-48 weeks old rat is not aged (40-50 years old???) when compared to human.

Apologies. This has been corrected. see page 21 line 529.

Although, it should be noted that this model is not age representative of the development of cardiorenal syndrome. In other words, it cannot be directly age-matched.

6. Introduction, second paragraph, reference #10. Please consider citing more updated statistics.

Apologies. This has been corrected. see line 81 in introduction

Reference: Roy et al. Working Group of the Endocrine Society of Bengal. Kidney Disease in Type 2 Diabetes Mellitus and Benefits of Sodium-Glucose Cotransporter 2 Inhibitors: A Consensus Statement. Diabetes Ther. 2020 Dec;11(12):2791-2827. doi: 10.1007/s13300-020-00921-y. Epub 2020 Oct 6. PMID: 33025397; PMCID: PMC7644753.

7. Page 11. SGLT trials (CREDENCE and DAPA-CKD) should not be bold.

Apologies. This has been corrected. Page 5 line 104-105

Reviewer 2:

A recent study demonstrated that accelerated cerebral vascular injury in diabetes is associated with vascular smooth muscle cell dysfunction (PMID: 32166556). Is it known if these features are exhibited by the GK rat?

This is outside the scope of the study. Unfortunately, in this study we did not investigate smooth muscle cell dysfunction. However, Sandu et al. (2000) demonstrated impaired vascular smooth muscle cell relaxation in GK rats (PMID: 11118023). There is no evidence that we are aware of that demonstrate accelerated cerebral vascular injury in the GK rat.

Similarly, can the authors comment on the plasma levels of mitokines FGF21, GDF15, and Humanin in the GK rat? Evidence has suggested those mitokines are elevated in type II diabetes and Alzheimer’s disease in comparison with healthy aging (PMID: 33131010)

This is outside the scope of our study. In this study we did not investigate the level of the mentioned mitokines within the plasma. Further, we did not see any significant changes in these mitokines within our proteomics analysis of the heart tissue. Finally, to our knowledge the plasma levels of the mentioned mitokines has not been investigated in the GK rat.

Grant requirements:

1) We note your current Financial Disclosure and Competing Interest read as follows:

“Studies were supported by research grants from the HSF Canada, and funds from the St Michaels Hospital Foundation “SCAR WARS” program. Dr Kim A Connelly is supported by a Merit Award from the Department of Medicine, University of Toronto.”

“KAC has received research grants to his institution from Astra Zeneca and Boehringer Ingelheim, received support for travel to scientific meeting from Boehringer Ingelheim and honoraria for speaking engagements and ad hoc participation in advisory boards from Astra Zeneca, Boehringer Ingelheim and Janssen. All other authors declare that they have no competing interests.”

We also note the following additional comments:

"Finally, Kim A. Connelly has received research grants to his institution from Astra Zeneca and

Boehringer Ingelheim, received support for travel to scientific meeting from Boehringer

Ingelheim and honoraria for speaking engagements and ad hoc participation in advisory boards

from Astra Zeneca, Boehringer Ingelheim and Janssen. However, this will have no-effect in

regard to data restriction and all pertinent data related to the study will be made available."

For the support regarding HSF Canada and St. Michael’s Hospital Foundation, please provide the following information:

a.) Any specific grant or award numbers associated with the support.

- **The HSF grant has an award number which has been added. The other grants are generic and do not have a specific name or award number, hence this has been left blank.**

b.) The names of the specific authors who received the support.

-**the grant is to Dr Kim A Connelly. The other authors did not receive funding to support this particular project.**

For the Merit Award from the Department of Medicine, University of Toronto, please provide the following information:

a.) Any specific grant or award numbers associated with the support.

-**The Merit award does not have a specific name or award number, hence this has been left blank.**

Please confirm whether there are any patents, products in development or marketed products associated with this research to declare.

**Reply:** There are no patents, products in development or marketed products associated with this research.

Please also confirm whether the support from Astra Zeneca, Boehringer Ingelheim, and Janssen, that is unrelated to the study, in any way alters your adherence to PLOS ONE policies on sharing data and materials.

**Reply:** There is no support from the aforementioned companies for this project. Dr Connelly has received monies for other projects. This is no way influences PLOS One policies and all data is freely available to be shared as there is no agreement with the above companies.
